# An update and systematic review on the treatment of primary dysmenorrhea

**DOI:** 10.5935/1518-0557.20180083

**Published:** 2019

**Authors:** Maedeh Sharghi, Shabnam Malekpour Mansurkhani, Damoon Ashtary Larky, Wesam Kooti, Mehdi Niksefat, Mohammad Firoozbakht, Masoud Behzadifar, Milad Azami, Karo Servatyari, Leila Jouybari

**Affiliations:** 1 Student Research Committee, Kurdistan University of Medical Sciences, Sanandaj, IR Iran; 2 Department of Biology, School of Science, Shiraz University, Shiraz, IR Iran; 3 Nutrition and Metabolic Diseases Research Center, Ahvaz Jundishapur University of Medical Sciences, Ahvaz, IR Iran; 4 Cellular and Molecular Research Center, Sabzevar University of Medical Sciences, Sabzevar, IR Iran; 5 Student Research Committee, Ahvaz Jundishapur University of Medical Sciences, Ahvaz, IR Iran; 6 Student Research Committee, Dezful University of Medical Sciences, Dezful, IR Iran; 7 Health Management and Economics Research Center, Iran University of Medical Sciences, Tehran, IR Iran; 8 Faculty of Medicine, Ilam University of Medical Sciences, Ilam, IR Iran; 9 Nursing Research Center, Golestan University of Medical Sciences, Gorgan, IR Iran

**Keywords:** primary dysmenorrhea, medicinal plants, chemical drugs

## Abstract

**Objectives:**

Primary dysmenorrhea is a painful uterine contraction caused by endometrial laceration. Drug therapies and complementary medicine have been used to treat dysmenorrhea**.** The aim of this study was to investigate and offer an updated perspective on the treatments for dysmenorrhea.

**Methods:**

The present study was conducted in accordance with the PRISMA checklist for systematic reviews and meta-analyses. The required information was collected based on searches for the following keywords: treatment, primary dysmenorrhea**,** medicinal plants, chemical drugs, and herbs. Searches were performed on databases Pubmed, Web of Sciences, Scopus, Iran medex, and SID by March 2018 to find literature in the English and Persian languages on this subject without a time limit.

**Results:**

This review included 17 papers, 10 of which on complementary medicine, three on drug therapies, and four on acupuncture and acupressure. The largest and smallest samples had 303 and 24 patients, respectively. Length of treatment ranged from one to six months and the measures most commonly used in the studies were the visual analogue scale and clinical efficacy. Reported complications included gastrointestinal events, nausea, vomiting, diarrhea, abdominal pain, and liver and kidney disorders.

**Conclusion:**

Medicinal plants, drugs, and acupressure seem to suppress pain by reducing the level of prostaglandins, mediating nitric oxide, increasing beta-endorphin levels, blocking the calcium channel, and enhancing circulatory flow through the uterine pathway. Further trials are required to confirm the benefits of the procedures described and ensure the absence of complications.

## INTRODUCTION

Primary dysmenorrhea is a painful uterine contraction caused by endometrial laceration. The pain caused by dysmenorrhea begins a few days before menstruation and persists for 48 to 72 hours. Cramping pain often reaches the thighs (Xu et al., 2017). Dysmenorrhea is one of the most common complaints of adolescents and mature women. It usually comes with a wide range of physical symptoms such as headaches, dizziness, fatigue, diarrhea, cramps, and sweating. Dysmenorrhea is the cause of one to three percent of the cases of absenteeism at school and work, which translates into a loss of 600 million hours a year and the equivalent to USD 2 billion in the United States. The condition is highly prevalent among women, with incidence ranging from 45% to 97% in groups of different ages and nationalities (Kim et al., 2017; Lee et al., 2016).

The causes of primary dysmenorrhea are still unclear, but one of the most accepted explanations is increased synthesis of prostaglandins, of which types E2 and F2α play a significant role in the development of ischemia and hypoxia, resulting in dysrhythmic uterine contractions and decreased blood flow (Ghafourian et al., 2015; Xu et al., 2017).

Drug therapies and complementary medicine are often used to treat dysmenorrhea (Chao et al., 2014; Hosseinlou et al., 2014; Kooti et al., 2014). According to the literature, NSAIDs and OCPs rank among the most frequently used medications. These drugs reduce pain by inhibiting the production and release of prostaglandins. However, long-term use of NSAIDs has been associated with side effects such as headache, dizziness, drowsiness, loss of appetite, nausea, vomiting, gastrointestinal bleeding, increased acute asthma, dysuria, and acne (Navvabi Rigi et al., 2012).

OCPs inhibit ovulation, reduce endometrial proliferation, and create an endocrine environment that mimics the early stages of the proliferative phase of the menstrual cycle, in which prostaglandin levels are at their lowest. Lower prostaglandin levels lead to fewer uterine cramps. In the realm of complementary medicine, methods resorting to herbs, yoga, relaxation, psychotherapy, massage, hypnosis, vitamins (E, B, C), and supplements (calcium and magnesium) as well as acupressure and acupuncture have been used (Chao et al., 2014; Hosseinlou et al., 2014; Lee et al., 2016; Xu et al., 2017). The herbs used more commonly to treat dysmenorrhea are chamomile, ginger, fennel, cinnamon, and aloe vera (Kim et al., 2017; Rahnama et al., 2012). Common treatments for dysmenorrhea are extensive and in some cases subject to restrictions. For example, NSAIDs are contraindicated for patients with digestive problems, while medicinal plants are not always readily available. This review aimed to investigate the progress reported in this field, shed light on newly developed methods to decrease pain in dysmenorrhea, and offer an update on dysmenorrhea therapy.

## MATERIALS AND METHODS

### Study design

The present study was conducted in accordance with the PRISMA (Preferred Reporting Items for Systematic Reviews and Meta-analyses) checklist and investigated recent relevant therapies for dysmenorrhea. Studies published in the English and Persian languages were included without a time limit.

### Search strategy

Searches were made on databases Pubmed, Web of Sciences, Scopus, Iran medex and SID for papers published by March 2018 using the following keywords: treatment, primary dysmenorrhea, medicinal plants, chemical drugs, and herbs.

### Inclusion Criteria

- Papers from randomized clinical trials
- The topic of the study was dysmenorrhea
- No infectious diseases, viruses, fungi, PID etc.

### Exclusion Criteria

- Inadequate sampling
- Limited sample size
- Infectious diseases, PID etc.

### Information Extraction

The abstracts of the studies were read by two expert reviewers and selected based on their inclusion and exclusion criteria. The reviewers resolved discrepancies together. The data sets extracted from the papers were processed after their quality had been confirmed. The information checklist for the study included the names of the authors, year of publication, sample size, method, study groups, main mechanism, and duration of treatment.

## RESULTS

A total of 17 papers were included, ten of which on complementary medicine, three on chemical drugs, and four on acupuncture and acupressure (Tables 1 and 2).

**Table 1:** New method of acupressure and drug therapies

Author- Year	Sample Size	Control Group	Case Group	Scale	Results
(Iacovides *et al.*, 2014)	24 women with dysmenorrhea	Placebo capsule (Gelatin, containing sugar) in 2 cycles	Diclofenac 150 mg during menstruation. in 2 cycles	VAS	Diclofenac decreased the severity of pain
(Dmitrovic *et al.*, 2013)	62 women with dysmenorrhea	100 mg placebo	100 mg single dose Vaginal Sildenafil citrate. First day of menstrual pain	VAS	Menstrual pain improved with vaginal sildenafil
(Liu *et al.*, 2011)	194 women with dysmenorrhea	Acupuncture and acupoint	Acupuncture in the acupoint region. Another group received acupuncture in an unrelated acupoint region. Once a day for the first 3 days of menstruation	VAS	Individuals offered acupuncture had fewer menstrual pain
(Cha & Sok, 2016)	91 students with dysmenorrhea	Only acupressure in the atria of the ear for the first 3 days	Acupuncture and acupressure in the ear region for the first 3 days of menstruation	VAS	Individuals offered ear acupressure had less dysmenorrhea, backache, and abdominal pain.
(Daniels *et al.*, 2009)	303 women with dysmenorrhea	_________	The first group was given celecoxib 400 mg single dose capsules; after 12 hours, they were given celecoxib 200 mg per day in 3 days of menstruation. The second group received naproxen 550 mg and a second dose of naproxen 550 mg 12 hours after the first dose, in 3 days of menstruation.	VAS	No significant difference was found between the two groups
(Kiran *et al.*, 2013)	35 women with dysmenorrhea	Received naproxen sodium 3 times a day from the second day before menstruation and were restarted on the third day of menstruation for one month	Acupuncture at HT 7. PC 6. LI 4 LI 10. SP 6. LR3 ST 36. GB 26. SP 15 3 times a day from the second day before menstruation; restarted on the third day of menstruation for 1 month	N/A. VAS	The severity of pain was reduced in the first group
(Yang *et al*., 2008)	120 women with dysmenorrhea	Received Indomethacin Treatment from 3 days before menstruation until the 5^th^ day of menstruation, 3 cycles	Received Superficial needling at sp 6 Treatment from 3 days before menstruation to the 5^th^ day of menstruation, 3 cycles	Symptom score + analgesic time. Clinical efficacy	The severity of pain in the first group was significantly decreased.

**Table 2:** Effective medicinal plants in primary dysmenorrhea treatment

***Foeniculum vulgar *Mill (Fennel)**	**Author**	**Research sample**	**Methodology**	**Scale**	**Results **
	(Torkzahrani *et al*., 2007)	90 students	5 capsules 46 mg daily containing extracts of fennel for the Case group and 5 placebo capsules for the control group	Verbal multidimensional scale	Significant decrease in pain severity between the case and control groups and lethargy range systemic symptoms of dysmenorrhea
	(Delaram & Forouzandeh, 2011)	60 students	30 drops of the fennel extract every 8 hours daily for the fennel group; control were given placebo following the same scheme.	VAS	Significant decrease in pain scores between case and control groups
	(Moslemi, *et al.*, 2012b)	65 single female students	46 Mg capsules of fennel and placebo 4 times daily for the fennel and placebo groups, respectively; and 100 IU of vitamin E capsules to the vitamin E group	VAS	Significant reduction in the mean duration of pain in the first and second months in the case group; significant reduction in the mean duration of pain in the second month of vitamin E; and significant reduction in the duration of pain between the three groups and decreased consumption of sedatives.
	(Ghodsi & Asltoghiri, 2014)	80 female students	30 mg of fennel every 4 hours, from 3 days before menstruation to 5 days after it given to the case group; no drugs given to controls.	VAS, McGill pain questionnaire	Decreases in nausea and weakness after 3 months and bleeding after 2 and 3 months; improved quality of life in months 1 and 3 in the case group
***Matricaria chamomilla*(Chamomile)**	(Yazdani *et al.*, 2004)	60 students	Five cycles of treatment: no medication given in the first; fennel was administered in the second and third cycles; chamomile was given in the fourth and fifth cycles.	Questionnaire	A significant reduction in the severity of abdominal and pelvic pain, fatigue and lethargy, depression and anger among the 16 symptoms of dysmenorrhea in comparison with the cycle without medication (Control)
	(Jenabi & Ebrahimzadeh, 2010)	80 students	A month before the intervention (control) and one and three months after the intervention, individuals were given two cups of chamomile herbal tea daily for three months.	(McGill Pain Questionnaire, Visual Analogue Scales for Anxiety, Perceived Stress Scale and The Psycho physiologic Life Adaptation Scale)	A significant reduction in pain intensity in the first and third months after the intervention compared to controls; and reduced levels of anxiety after a month compared to controls.
	(Modarres *et al.*, 2011)	80 students	For two consecutive cycles, a group was given mefenamic acid 250 mg and another group was given 400 mg of chamomile.	VAS	Mean pain severity decreased in both groups after two treatment cycles; significant decreases were seen only in the chamomile group.
***Zataria multiflora***	(Iravani, 2009)	108 students	25 drops every four hours of thyme 1% or thyme 2%.	Multi Dimensional System, VAS	Significant decreases in pain scores between the case and control groups
	(Direkvand-Moghadam & Khosravi, 2012)	120 students	5ml thyme extract four times a day to a group and 3 tablets containing 400mg ibuprofen 3 times a day to the other group.	VAS	Pain intensity decreased in the two groups. No significant differences between groups. The two groups had shorter duration of pain.
	(Salmalian *et al.*, 2014)	84 students	200 mg ibuprofen and 25 drops of essential oil to the ibuprofen group; 25 drops of essential oil and 200 mg of thyme to the thyme group; and placebo capsule and 25 drops of essential oil to controls.	VAS	Pain intensity decreased in the three groups. No significant difference between the ibuprofen and thyme groups. Significant difference between the ibuprofen and thyme groups and controls.

In the field of complementary medicine, five studies looked into *Foeniculum vulgar* Mill (Fennel) (2014-2007), three into *Matricaria chamomilla* (Chamomile) (2010-2004), and three into *Zataria multiflora* (2014-2008). In the field of drug therapy, one study compared celexcib capsules with naproxen (2009); one compared vaginal sildenafil citrate with vaginal placebo (2013); and a study compared oral mefenamic acid with placebo capsule with sugar (2013).

The largest and smallest samples had 303 and 24 patients, respectively. Controls were given placebo capsules (containing sugar or starch), vaginal tablets (placebo), fenbid pills, ibuprofen tablets, vitamin E, mefenamic acid, essential oil, indomethacin or naproxen, while individuals in the case group were given oral diclofenac, vaginal sildenafil citrate, oral celecoxib, fennel capsules or drops, chamomile capsules or *Zataria multiflora* drops.

Length of treatment ranged from one to six months and the measures most commonly used in the studies were the visual analogue scale and clinical efficacy. Reported complications included gastrointestinal events, nausea, vomiting, diarrhea, abdominal pain, and liver and kidney disorders.

## DISCUSSION

This systematic review comprised studies on the use of complementary medicine, drug therapies, and acupressure to treat dysmenorrhea. A total of 17 clinical trials were included. Ten studies on complementary medicine looked into the use of plants such as fennel, chamomile, and *Zataria multiflora*. Fennel belong to the Umbelliferae family and the main ingredients found in it are anethole, limonene, and fenchone (Taherian et al., 2007). Its roots, leaves, and fruits have anti-inflammatory and anti-spasmodic properties (Lim, 2012). It is also a known carminative, diuretic, and laxative with anti-ulcerative and antioxidant properties in digestive injuries, employed in the treatment of neurological disorders (Birdane et al., 2007).

The fruit of the fennel plant is a source of anethole (Taherian et al., 2007). Anethole is very similar to dopamine. It binds to the dopamine receptor and inhibits pain and suppresses contractions induced by oxytocin, prostaglandin E2, acetylcholine, and histamines. Fennel, in addition to having 10-12% oil, contains small amounts of sugar and mucilage. Fennel essence has phenolic ethers, a relevant factor in its medicinal properties (Taherian et al., 2007). In general, fennel neutralizes oxytocin and prostaglandin-induced spasms and induces menstrual bleeding in shorter intervals, which by its turn decreases pain (Modaress Nejad et al., 2006). Fennel has also been used in Chinese and European traditional medicine (Birdane et al., 2007). In the studies conducted by Torkzahrani et al. (2007), Delaram & Forouzandeh (2011) and Ghodsi & Asltoghiri (2014) on the therapeutic effects of fennel on dysmenorrhea, the plant was characterized as a possible therapeutic agent for lack of complications and for its analgesic effects (Moslemi et al., 2012a; Delaram & Forouzandeh, 2011; Ghodsi & Asltoghiri, 2014).

Chamomile flowers contain some 120 chemical compounds including flavonoids, glycoside 3%, azolin, apigenin, and methoxycoumarin (Letchamo & Marquard, 1993). Flavonoids are the main agents responsible for antispasmodic and antioxidant effects (Ranjbar et al., 2015). The essential oils of this plant, especially bisabolol and karmasolen, have anti-inflammatory effects (Khatami Sabzevar et al., 2017). Chamomile is a plant with analgesic, antipyretic, antirheumatic, anti-inflammatory, carminative and sedative properties known for increasing menstrual blood flow (Salamon, 1992). Effects have been reported on the treatment of migraines and muscle soreness (Abdollahi Arjenki, 2016). Yazdani et al. (2004), Jenabi & Ebrahimzadeh (2010), and Modarres et al. (2011) looked into the therapeutic effects of chamomile and characterized it as a possible treatment for dysmenorrhea.

*Zataria multiflora* contains thymol, which inhibits contractions caused by cell scaling and blocks the calcium channel, thus directly affecting pain receptors and eventually inhibiting the release of prostaglandins. Iravani (2009) and Salmalian et al. (2014) concluded that *Zataria multiflora* is a suitable drug to treat individuals with dysmenorrhea due to the absence of side effects. In a review, Zu et al. found that some herbs were more effective than placebo, thermotherapy, and acupuncture, although sometimes additional treatment beyond drug therapy was needed (Salehian et al., 2011).

In the field of drug therapies, three papers on NSAIDs were reviewed. NSAIDs disrupt the conversion of arachidonic acid into endoperoxides (COX) and thereby inhibit the production and release of prostaglandins (Xu et al., 2017). Pain is thus neutralized, but long-term consumption of steroids has been associated with side effects such as headache, dizziness, drowsiness, loss of appetite, nausea, vomiting, gastrointestinal bleeding, acute asthma, dysuria, and acne (Salmalian et al., 2014). People with digestive problems should only take the medication with specialist advice and under supervision (Kalpana et al., 2014). In a study, Daniels et al. (2009) concluded that celecoxib had analgesic effect, while cyclooxygenase-2 inhibition neutralized menstrual pain. Iacovides et al. (2014) suggested NSAIDs were effective in the management of dysmenorrhea. Dmitrovic et al. (2013) found that sildenafil citrate increased nitric oxide and decreased phosphodiesterase type 5 (PDE5) levels, thereby neutralizing menstrual pain.

Dysmenorrhea has been causally linked to decreased progesterone steroid hormone levels in the luteal phase, a condition connected to lower levels of lysosomal enzymes and the ensuing release of endometrial phospholipase A2. These events lead to increased levels of prostaglandins responsible for the contraction of the uterus and arteries, ultimately causing ischemia and pain in the womb (Barbieri & Ryan, 1999).

A meta-analysis by Xu et al. (2017) looked into 19 studies on acupressure. Acupuncture is believed to stimulate receptors and neural pathways that block pain impulses by interacting with mediators such as serotonin and endorphins (Xu et al., 2017). Acupuncture has been introduced as an effective non-pharmacological treatment for dysmenorrhea to decrease absenteeism in the workplace (Bahrami-Taghanaki et al., 2017). Acupressure is another non-pharmacological method used as a means to alleviate primary dysmenorrhea. In acupressure, pressure by a hand, finger or thumb is applied on the same stimulation points used in acupuncture (Akbarzade et al., 2011). Acupressure uses touch to balance two energy flows of the human body known as kai (Sadat et al., 2015). Kai is the vital energy manifested through organic functions. Disease takes over when kai cannot flow properly through the body. Theoretically, the cause of primary dysmenorrhea is a shortage or decline of energy in the uterus, and the treatment for painful menstrual bleeding requires the modulation of the flow of energy and blood and the regulation of the organs of the body, particularly the liver, spleen, and kidneys (Akhavan Amjadi et al., 2015). Sinjiao (sp6) or the three-channel connection (between the spleen, liver, and kidneys) is one of the most important stimulation points in acupressure. In it, four fingers are placed above the ankle behind the posterior margin of the tibia, to stimulate one of the internal branches that passes through the womb (Rakhshekhorshid et al., 2013). This point is widely used in the treatment of gynecologic disorders, genitourinary disorders, gastrointestinal problems, weakness, low blood pressure, anesthesia during pelvic surgery, and in painless labor (Wang et al., 2004; Yu et al., 2010). The meta-analysis by Xu et al. (2017) compared between a variety of acupressure methods and drug therapies and found that acupressure might be a good therapy for dysmenorrhea due to the lack of side effects.

## CONCLUSION

Medicinal plants, drugs, and acupressure seem to suppress pain by reducing the level of prostaglandins, mediating nitric oxide, increasing beta-endorphin levels, blocking the calcium channel, and enhancing circulatory flow through the uterine pathway. Further trials with larger populations, longer durations, featuring comparisons with safe drugs and accurate descriptions of the involved molecular mechanisms are required to confirm the benefits of the procedures described and ensure the absence of complications. The conclusions presented herein are also affected by the fact that some of the methods were analyzed by only a handful of studies.
